# In Vitro Evaluation of Redox-Associated Responses Induced by Mud Extract in L929 and RAW 264.7 Cells

**DOI:** 10.3390/antiox15040448

**Published:** 2026-04-02

**Authors:** Hyeong Ho Kim, Sung Hun Jang, Jae-Sik Jeon, Jae Kyung Kim

**Affiliations:** 1 Department of Biomedical Laboratory Science, College of Health Sciences, Dankook University, Cheonan-si 31116, Republic of Korea; hohyeongkim@dankook.ac.kr (H.H.K.); 12170376@dankook.ac.kr (J.-S.J.); 2 Department of Medical Laser, Graduate School of Medicine, Dankook University, Cheonan-si 31116, Republic of Korea; shj5708@dankook.ac.kr

**Keywords:** mud extract, oxidative stress, antioxidant enzymes, Nrf2/HO-1 pathway

## Abstract

While natural muds are widely used in traditional balneotherapy and dermatological applications, the cellular basis of their redox-related effects remains insufficiently defined. In this study, we evaluated the effects of a mineral-rich mud extract on L929 fibroblasts and RAW 264.7 macrophages. This study was designed as an initial in vitro exploratory investigation to evaluate the cellular responses induced by a complex mud-derived material containing multiple inorganic components under standardized extract conditions. The mud extract showed no overt cytotoxicity up to 1000 μg/mL under the tested conditions. Intracellular reactive oxygen species (ROS) levels remained near baseline across the measured time points, with limited cell type-dependent variation. In parallel, antioxidant-related responses were observed primarily in RAW 264.7 cells, including a transient early increase in superoxide dismutase (SOD)-associated activity and subsequent increases in catalase (CAT) and glutathione peroxidase (GPx) activities. Reverse transcription-quantitative polymerase chain reaction (RT-qPCR) analysis further showed dose-dependent upregulation of nuclear factor erythroid 2-related factor 2 (Nrf2) and heme oxygenase-1 (HO-1) transcripts, particularly in RAW 264.7 cells. Collectively, these findings suggest that the mud extract is associated with coordinated antioxidant-related responses under non-cytotoxic conditions. However, because the present study was conducted in two murine cell lines and relied partly on assay systems potentially susceptible to matrix effects, the results should be interpreted as supportive of redox-associated modulation rather than definitive proof of a therapeutic mechanism. Furthermore, these findings should be interpreted as preliminary evidence obtained from a standardized aqueous extract system and not as definitive proof of component-specific mechanisms or direct applicability.

## 1. Introduction

Oxidative stress is a fundamental biological process implicated in cellular damage, aging, and the pathogenesis of numerous diseases [1,2,3,4]. It arises primarily from an imbalance between the generation of reactive oxygen species (ROS) and the capacity of endogenous antioxidant defense systems to neutralize them [5]. To maintain redox homeostasis, cells rely on tightly regulated antioxidant enzymes, including superoxide dismutase (SOD), catalase (CAT), and glutathione peroxidase (GPx) [6,7]. Disruption of this balance can result in oxidative injury, mitochondrial dysfunction, and activation of inflammatory signaling pathways [8,9]. Furthermore, oxidative stress is intrinsically linked to cellular metabolic homeostasis, as reactive oxygen species (ROS) are both by-products of mitochondrial respiration and active modulators of metabolic flux [10,11]. Recent studies suggest that maintaining the redox-metabolic interface is crucial for preventing metabolic reprogramming, where cells shift energy resources to sustain antioxidant defenses under environmental burden [12,13].

In recent years, increasing attention has been directed toward natural materials as potential modulators of cellular redox balance [14,15,16]. Unlike classical antioxidant compounds that act primarily as direct radical scavengers, mineral-rich natural materials may influence endogenous antioxidant systems and redox-sensitive cellular processes [17,18,19]. Through such modulation, these materials may support balanced antioxidant regulation rather than inducing excessive or suppressive antioxidant effects [20,21,22].

Mud is a natural material enriched with diverse minerals (e.g., zinc, copper, manganese, magnesium, and selenium) and bioactive components and has long been utilized in therapeutic and cosmetic applications [23,24,25,26]. Given that many of these trace elements serve as essential cofactors for primary antioxidant enzymes, mud extract may represent a potential modulator of cellular redox balance [27,28,29]. Despite its widespread use, the cellular-level mechanisms underlying its potential redox-modulatory properties remain insufficiently characterized [24,30,31]. Limited information is available regarding how mud extract influences endogenous antioxidant-related activity and intracellular ROS regulation in mammalian cells [32,33,34].

For complex natural materials such as mud, an extract-based approach can serve as a practical starting point for controlled in vitro investigations, allowing the assessment of overall cellular responses under standardized conditions. However, this approach has inherent limitations, as it does not fully resolve the contributions of individual components or the complexity of the original material. Therefore, the present study aimed to explore redox-associated cellular responses induced by a standardized aqueous mud extract under non-cytotoxic conditions in two biologically distinct cell models, L929 fibroblasts and RAW 264.7 macrophages. To achieve this, we evaluated cell viability, intracellular ROS levels, antioxidant enzyme activities (SOD, CAT, and GPx), and the expression of antioxidant-related genes (Nrf2 and HO-1). This study was designed as an exploratory investigation to provide foundational insight into redox-associated cellular responses induced by mud-derived materials in controlled in vitro settings.

## 2. Materials and Methods

### 2.1. Preparation of Mud Extract

An aqueous extract of Boryeong mud was prepared through a multi-step process involving pre-treatment, desalting, and extraction. Initially, raw mud was suspended in purified water at a 1:5 (*w*/*w*) ratio, agitated at room temperature (RT) for 40–80 min, and allowed to sediment. The supernatant was then discarded, and this washing cycle was repeated three times. For the desalting phase, the pre-treated mud was resuspended in purified water (1:5, *w*/*w*), stirred for 40–60 min at RT, and left to settle at 4 °C for 12–20 h.

Subsequently, the extraction was performed by mixing the desalted mud with purified water at a 1:2 (*w*/*w*) ratio. After agitating for 40–60 min at RT, the mixture was incubated at 4 °C for 12–20 h to facilitate sedimentation. The resulting supernatant was collected and subjected to drum filtration to remove coarse suspended materials, followed by secondary vacuum filtration through filter paper. Finally, the filtrate was supplemented with phenoxyethanol and 1,3-butylene glycol as preservatives and filter-sterilized. This refined mud extract was utilized as the stock solution for all subsequent experiments. This extraction protocol was designed to obtain a relatively consistent aqueous fraction suitable for in vitro evaluation, rather than to fully reproduce the physicochemical complexity of the original raw mud.

### 2.2. Elemental Composition Analysis and Dose Preparation

The elemental composition of the mud extract was determined using inductively coupled plasma–optical emission spectrometry (ICP-OES). Briefly, the aqueous extract underwent acid digestion to solubilize inorganic elements, which were subsequently quantified. Elemental concentrations were expressed as weight percentages (wt.%) relative to the dried-extract equivalents, which were determined by lyophilizing a known volume of the liquid extract. For reference, oxide contents were calculated from elemental concentrations using standard stoichiometric conversion factors. This analysis aimed to characterize bioaccessible inorganic components potentially involved in the observed cellular redox-modulatory effects.

To ensure precise dosing in cell culture experiments, the concentration of the mud extract was standardized based on its dry residue. According to the certificate of analysis, the dry residue of the stock extract was 2.0% (*w*/*w*; 5 g, 160 °C, 2 h). To improve experimental consistency, the extract was standardized based on dried-residue equivalents, and all treatment concentrations were defined accordingly. In addition, ICP-OES analysis was performed to characterize the inorganic composition of the extract. Despite these efforts, it should be noted that extract-based systems inherently involve compositional variability and do not allow precise attribution of observed effects to individual components. Assuming a solution density of 1.0 g/mL, the stock extract contained approximately 20 mg/mL of dried-residue equivalents. Accordingly, working concentrations of 10, 100, and 1000 μg/mL were prepared by diluting the stock solution in complete culture medium at ratios of 1:2000, 1:200, and 1:20 (*v*/*v*), respectively.

### 2.3. Cell Culture

L929 mouse fibroblasts and RAW 264.7 mouse macrophages were obtained from the Korean Cell Line Bank (Seoul, Republic of Korea). L929 cells were cultured in Roswell Park Memorial Institute (RPMI) 1640 medium (Gibco, Grand Island, NY, USA), while RAW 264.7 cells were maintained in Dulbecco’s Modified Eagle’s Medium (DMEM; Gibco). Both media were supplemented with 10% fetal bovine serum (FBS; Gibco, Grand Island, NY, USA) and 1% penicillin–streptomycin (Gibco, Grand Island, NY, USA). The cells were incubated at 37 °C in a humidified atmosphere containing 5% CO_2_. For all experiments, cells were seeded and allowed to adhere for 24 h before being subjected to mud extract treatment.

### 2.4. Treatment and Sampling

Following the 24 h attachment period, the culture medium was replaced with fresh medium containing mud extract at concentrations of 10, 100, and 1000 μg/mL (dried-residue equivalents). The concentration range of 10, 100, and 1000 μg/mL was selected based on a logarithmic scale to comprehensively screen the dose-dependent effects of the mud extract. This wide range allows for the identification of potential biological thresholds and the evaluation of dose-dependent responses across redox-related endpoints.

Cells maintained in complete medium without mud extract served as the untreated control (Ctrl). To evaluate time-dependent redox-related changes, samples were harvested at multiple time points. Specifically, intracellular ROS levels were monitored at 1, 6, and 24 h to capture the kinetic profile of the oxidative response. For longer-term biological effects, cell viability and antioxidant enzyme activities were assessed at 24 h and 48 h post-treatment. All experiments were performed in at least triplicate to ensure statistical reliability.

### 2.5. Cell Viability Assay

Cell viability was evaluated using a water-soluble tetrazolium salt (WST) assay (WST-8 Cell Counting Kit; Biomax, Guri, Republic of Korea) to assess the potential cytotoxicity of the mud extract. L929 and RAW 264.7 cells were seeded in 96-well plates at a density of 1 × 10^4^ cells per well and allowed to adhere for 24 h. The cells were then treated with mud extract at the indicated concentrations (10, 100, and 1000 μg/mL) and incubated for 24 h and 48 h. At each time point, 10 μL of the WST-8 reagent was added to each well. The plate was then incubated for 3 h at 37 °C in a humidified 5% CO_2_ atmosphere. Prior to reading, the microplate was briefly shaken, and the absorbance was measured at 450 nm using a microplate reader (Tecan, Männedorf, Switzerland), and cell viability was calculated as a percentage relative to the untreated Ctrl group. To account for potential optical interference by mineral components or particulate suspensions in the mud extract, cell-free background wells (containing culture medium, mud extract, and WST reagent) were included for each concentration and time point. These background absorbance values were subtracted from the corresponding cell-containing wells prior to calculating viability.

To completely rule out any residual optical interference from the extract and to orthogonally validate the WST assay results, absolute cell viability was further evaluated using the trypan blue exclusion assay. Briefly, cells were seeded and treated with the mud extract for 24 h and 48 h under identical conditions. Following the incubation periods, the cells were harvested and stained with trypan blue solution. The total number of viable cells per well was then directly quantified using a TC20 Automated Cell Counter (Bio-Rad, Hercules, CA, USA).

All measurements for both assays were performed in triplicate to ensure that the experimental conditions did not induce cytotoxic effects that could confound the interpretation of subsequent redox-related assays.

### 2.6. Measurement of Intracellular Reactive Oxygen Species (ROS)

Intracellular ROS levels were assessed using an OxiTec Cellular ROS Detection Kit-H2DCFDA (Biomax). Cells were incubated with 10 μM 2′,7′-dichlorodihydrofluorescein diacetate (DCFH-DA) for 30 min at 37 °C in the dark, followed by two washes with phosphate-buffered saline (PBS) to remove extracellular dye. Fluorescence intensity was measured at excitation/emission wavelengths of 485/535 nm using a fluorescence microplate reader (Tecan).

As specified in Section 2.4, ROS measurements were performed at 1, 6, and 24 h post-treatment to capture the kinetic profile of the intracellular oxidative response. The results were expressed as percentages relative to the untreated Ctrl group. The data are presented as mean ± SD from three independent experiments. To control for potential optical artifacts, cell-free wells containing culture medium supplemented with mud extract were evaluated under identical conditions to assess auto-fluorescence or quenching effects; these values were used for background correction.

It should be noted that DCFH-DA detects general intracellular oxidative activity and does not discriminate among specific reactive species (e.g., H_2_O_2_, ^•^OH or ONOO^−^). Therefore, the data reflect relative changes in the overall oxidative signal rather than the direct quantification of individual radical species.

### 2.7. Measurement of Antioxidant Enzyme Activities (SOD, CAT, and GPx)

To evaluate the enzymatic antioxidant defense system, the activities of SOD, CAT, and GPx were quantified using intracellular cell lysates. At designated time points (1, 6, and 24 h), cells were harvested and lysed according to the manufacturer’s instructions, and total protein concentrations were determined using a bicinchoninic acid (BCA) protein assay (Thermo Fisher Scientific, Waltham, MA, USA) to normalize enzymatic activity. Equal amounts of protein (20 μg) were used for each reaction to ensure consistency across samples.

SOD-associated antioxidant activity was measured using a SOD Assay Kit (Biomax), which monitors the inhibition of WST-1 reduction by superoxide anions generated via the xanthine oxidase system, with absorbance measured at 450 nm using a microplate reader (Tecan).

CAT activity was determined using the OxiTec™ Catalase Assay Kit (Biomax) based on the decomposition of hydrogen peroxide (H_2_O_2_). In this assay, the catalase in the sample reacts with H_2_O_2_, and the remaining H_2_O_2_ is subsequently reacted with an Oxi-Probe and horseradish peroxidase (HRP) to produce a colorimetric signal measured at 570 nm. One unit of CAT was defined as the amount of enzyme that decomposes 1 μmol of H_2_O_2_ per minute at pH 7.0 and 25 °C.

GPx activity was quantified using the EZ-Glutathione Peroxidase Assay Kit (DoGenBio, Seoul, Republic of Korea) by monitoring the rate of reduced nicotinamide adenine dinucleotide phosphate (NADPH) consumption at 340 nm. Since GPx activity is proportional to the decrease in NADPH absorbance as oxidized glutathione (GSSG) is converted back to reduced glutathione (GSH) by glutathione reductase, one unit of GPx was defined as the amount of enzyme that oxidizes 1 nmol of NADPH to nicotinamide adenine dinucleotide phosphate (*NADP*^+^) per minute at 25 °C.

To rigorously account for potential optical interference or non-enzymatic catalytic reactions caused by the mineral components of the mud extract, cell-free background controls were included for all enzymatic assays. These controls, containing only the mud extract and assay reagents without cell lysates, were processed under identical conditions, and their absorbance values were subtracted from the experimental values to ensure the data specifically reflected intracellular enzymatic activities. All results were expressed as units per milligram of protein (U/mg protein) and further normalized to the untreated Ctrl (100%) for comparative analysis.

### 2.8. Quantitative Real-Time PCR Analysis of Antioxidant Gene Expression Induced by Mud Extract in L929 and RAW 264.7 Cells

Total RNA was extracted from L929 fibroblasts and RAW 264.7 macrophages following treatment with mud extract using a commercial RNA isolation reagent (TRIzol; Thermo Fisher Scientific, Waltham, MA, USA), according to the manufacturer’s instructions. The concentration and purity of RNA were assessed spectrophotometrically, and equal amounts of RNA were reverse-transcribed into complementary DNA (cDNA) using the PrimeScript RT reagent kit (Takara Bio Inc., Shiga, Japan).

Quantitative real-time PCR (qPCR) was performed using a SYBR Green-based detection system (PowerUp SYBR Green Master Mix; Thermo Fisher Scientific, Waltham, MA, USA) on a real-time PCR instrument (QuantStudio 3 Real-Time PCR System; Thermo Fisher Scientific, Waltham, MA, USA) under the manufacturer’s recommended cycling conditions. The mRNA expression levels of nuclear factor erythroid 2-related factor 2 (Nrf2) and heme oxygenase-1 (HO-1) were analyzed, with glyceraldehyde 3-phosphate dehydrogenase (GAPDH) used as the internal reference gene. Relative gene expression was calculated using the comparative 2^−ΔΔCt^ method.

The primer sequences used were as follows:

**Nrf2**, forward 5′-TCTTGGAGTAAGTCGAGAAGTGT-3′ and

reverse 5′-GTTGAAACTGAGCGAAAAAGGC-3′;

**HO-1 (Hmox1)**, forward 5′-AAGCCGAGAATGCTGAGTTCA-3′ and

reverse 5′-GCCGTGTAGATATGGTACAAGGA-3′;

**GAPDH**, forward 5′-AGGTCGGTGTGAACGGATTTG-3′ and

reverse 5′-TGTAGACCATGTAGTTGAGGTCA-3′. All reactions were performed in triplicate.

Amplification specificity was confirmed by melt-curve analysis, and no-template controls were included to verify the absence of contamination. Primer specificity was further verified in silico against the mouse genome, and amplification efficiency was confirmed to be within an acceptable range.

### 2.9. Statistical Analysis

All quantitative results are presented as the mean ± standard deviation (SD) from three independent experiments (*n* = 3) to confirm reproducibility. Statistical evaluations were conducted utilizing IBM SPSS Statistics software (version 29.0; IBM Corp., Armonk, NY, USA). To determine the statistical significance of differences among multiple treatment groups, a one-way analysis of variance (ANOVA) was performed, followed by Tukey’s post hoc test for multiple comparisons. For data involving both multiple concentrations and time points, statistical comparisons were made within each time interval to identify dose-dependent effects. A *p*-value of less than 0.05 was considered to indicate statistical significance.

## 3. Results

### 3.1. Elemental Composition of Mud Extract

The elemental composition of the aqueous mud extract, determined by ICP-OES, revealed a diverse profile of inorganic constituents (Table 1). Aluminum (Al) and iron (Fe) were identified as the most abundant elements, comprising 7.59 wt.% and 2.87 wt.% of the dried extract, respectively. Essential physiological electrolytes, including potassium (K) and sodium (Na), were also present at significant levels (2.52 wt.% and 1.87 wt.%, respectively), followed by magnesium (Mg, 0.85 wt.%) and calcium (Ca, 0.79 wt.%). Notably, the trace element manganese (Mn) was detected at 0.032 wt.%.

Importantly, this elemental profile represents the extractable inorganic fraction obtained under aqueous conditions, rather than the bulk mineral matrix of the raw mud. Therefore, these results reflect the bioaccessible components that are readily solubilized and available to interact with cellular systems.

### 3.2. Effects of Mud Extract on Cell Viability

**Figure 1:**
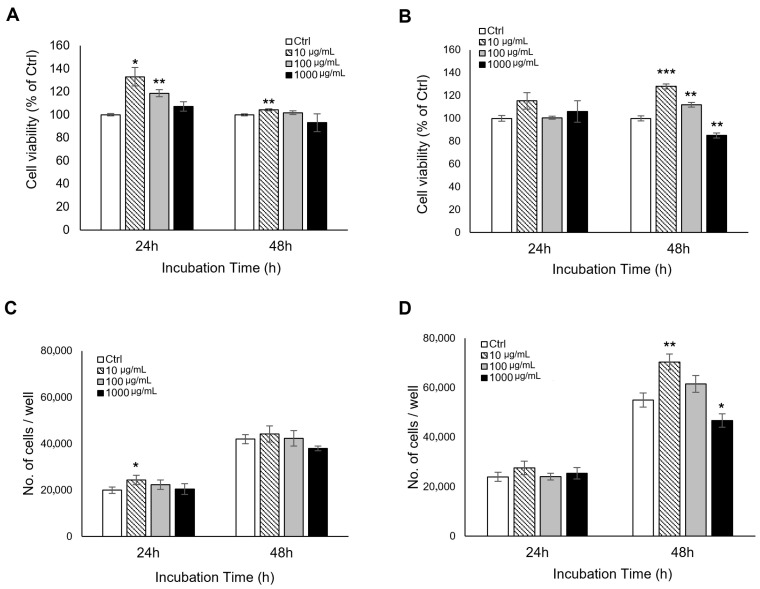
Effects of mud extract on the viability and proliferation of L929 and RAW 264.7 cells. Left panels (**A**,**C**) represent L929 fibroblasts, and right panels (**B**,**D**) represent RAW 264.7 macrophages. (**A**,**B**) Cell viability was evaluated using the WST assay after treatment with indicated concentrations (10, 100, and 1000 μg/mL) of mud extract for 24 and 48 h. (**C**,**D**) To rule out potential optical interference from the extract, cell proliferation was orthogonally validated using the trypan blue exclusion assay via direct cell counting. The data are presented as the mean ± standard deviation (SD) of three independent experiments (*n* = 3). * *p* < 0.05, ** *p* < 0.01, and *** *p* < 0.001. Asterisks indicate statistically significant differences compared to the respective control (Ctrl) group at each time point. Bars without asterisks indicate no significant difference (*p* > 0.05) compared to the Ctrl.

To evaluate the cytocompatibility of the mud extract, cell viability was initially assessed using the WST assay. In L929 fibroblasts, treatment with the extract for 24 h resulted in an increase in WST absorbance compared to the control, while no significant reductions were observed at 48 h across all concentrations (Figure 1A). To orthogonally validate these findings and completely rule out potential optical interference from the mineral-rich extract, the trypan blue exclusion assay was performed. Direct cell counting revealed that L929 cells proliferated normally over 48 h; even at the highest concentration (1000 μg/mL), the absolute cell numbers remained comparable to the control group, showing no statistically significant reduction (Figure 1C). Similarly, in RAW 264.7 macrophages, the mud extract did not induce significant changes in cell viability at 24 h across all tested concentrations (Figure 1B). At 48 h, a dose-dependent trend was observed; while lower concentrations slightly increased viability, the 1000 μg/mL treatment resulted in a moderate reduction to approximately 85% of the control. Crucially, this metabolic trend was closely mirrored by the orthogonal trypan blue assay (Figure 1D), which confirmed a consistent pattern in actual cell numbers. Collectively, both the WST and trypan blue assays consistently demonstrated that the mud extract does not induce severe cytotoxic effects in either L929 or RAW 264.7 cells at concentrations up to 1000 μg/mL under the experimental conditions. These findings established a largely non-cytotoxic concentration range for subsequent redox-related analyses under the present experimental conditions.

### 3.3. Effects of Mud Extract on Intracellular ROS Generation

**Figure 2:**
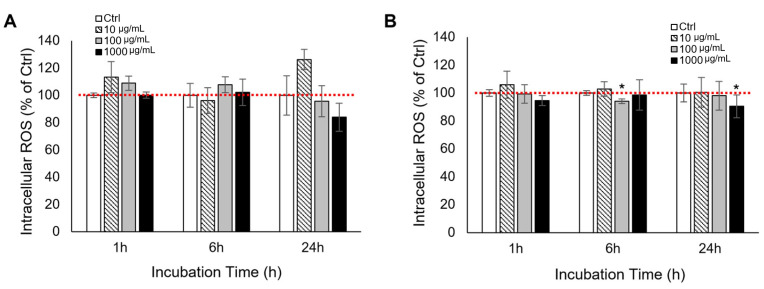
Kinetic profiles of intracellular ROS generation in L929 and RAW 264.7 cells. Intracellular ROS levels were measured using the DCFH-DA assay at 1, 6, and 24 h after treatment with various concentrations (10, 100, and 1000 μg/mL) of the mud extract. The red dotted line represents the baseline level (100%) of the untreated control (Ctrl). (**A**) L929 fibroblasts and (**B**) RAW 264.7 macrophages. The data are expressed as a percentage of the untreated control (Ctrl) after background subtraction. All values represent the mean ± SD (*n* = 3). Asterisks indicate significant differences compared to the control (* *p* < 0.05).

To evaluate early redox-related responses, intracellular ROS levels were measured in L929 fibroblasts and RAW 264.7 macrophages at 1, 6, and 24 h after treatment. Overall, mud extract did not induce marked increases in ROS levels across the tested concentrations. In L929 fibroblasts, no statistically significant deviations from the untreated control were observed at any time point. In RAW 264.7 macrophages, ROS levels also remained close to control values, although modest reductions were observed at 6 h (100 μg/mL) and 24 h (1000 μg/mL) (* *p* < 0.05) (Figure 2). These findings indicate that, under the present conditions, mud extract did not trigger an overt oxidative burst and was associated with near-baseline intracellular ROS levels.

### 3.4. Modulation of SOD, CAT, and GPx Activities by Mud Extract in L929 and RAW 264.7 Cells

**Figure 3:**
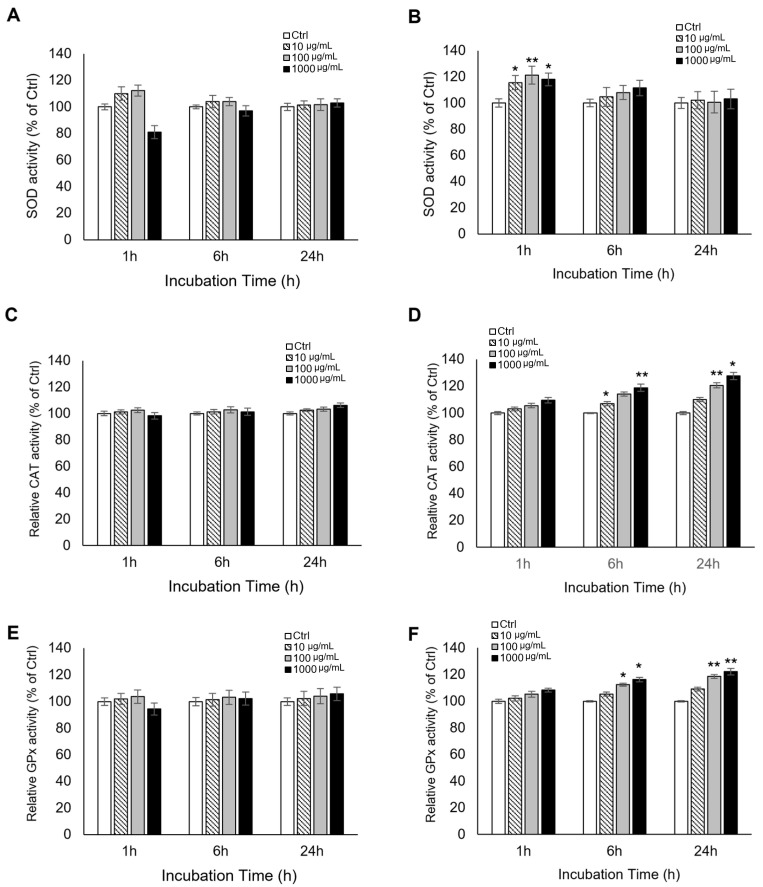
Kinetic profiles of antioxidant enzyme activities in L929 and RAW 264.7 cells. The relative activities of superoxide dismutase (SOD), catalase (CAT), and glutathione peroxidase (GPx) were quantified at 1, 6, and 24 h after treatment with various concentrations (10, 100, and 1000 μg/mL) of the mud extract. Left panels (**A**,**C**,**E**) represent L929 fibroblasts, and right panels (**B**,**D**,**F**) represent RAW 264.7 macrophages. (**A**,**B**) SOD-associated activity was measured by monitoring the inhibition of WST-1 reduction. (**C**,**D**) CAT activity was determined by measuring the decomposition of H_2_O_2_ through a HRP-linked colorimetric assay. (**E**,**F**) GPx activity was quantified by monitoring the rate of NADPH consumption at 340 nm. To eliminate potential optical interference from the mineral components of the extract, background absorbance from cell-free blanks was subtracted for each concentration across all assays. The data are expressed as a percentage of the untreated control (Ctrl) and represented as the mean ± SD (*n* = 3). Asterisks indicate significant differences compared to the Ctrl (* *p* < 0.05, ** *p* < 0.01). Bars without asterisks indicate no significant difference (*p* > 0.05) compared to the Ctrl.

To investigate antioxidant-related enzymatic responses, the relative activities of SOD, CAT, and GPx were evaluated. In L929 fibroblasts, the activities of all three enzymes remained largely stable across the tested concentrations and time points (Figure 3A,C,E). In RAW 264.7 macrophages, a distinct time-dependent response pattern was observed (Figure 3B,D,F). SOD-associated activity increased transiently at 1 h, reaching approximately 120% of the control at 100 μg/mL (** *p* < 0.01), and then returned toward baseline by 6 h. CAT and GPx activities showed increases at later time points, with the most prominent changes observed at 24 h. These findings suggest that mud extract is associated with a temporally differentiated antioxidant-related response in RAW 264.7 cells, whereas L929 fibroblasts showed comparatively limited changes under the same conditions. However, these findings should be interpreted as indicative of antioxidant-related responses rather than definitive evidence of direct enzymatic induction or a fully established antioxidant cascade.

### 3.5. RT-qPCR Analysis of Antioxidant-Related Gene Expression After Mud Extract Treatment in L929 and RAW 264.7 Cells

**Figure 4:**
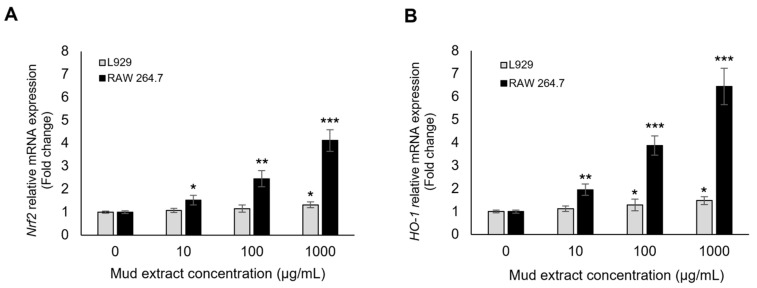
Transcriptional induction of the Nrf2/HO-1 signaling pathway by mud extract. Relative mRNA expression levels of (**A**) Nrf2 and (**B**) HO-1 were determined by RT-qPCR in L929 and RAW 264.7 cells 12 h after treatment with various concentrations (0, 10, 100, and 1000 μg/mL) of mud extract. Values were normalized to GAPDH and are expressed as fold changes relative to the untreated control (0 μg/mL). While L929 fibroblasts showed a modest increase in antioxidant gene expression, RAW 264.7 macrophages exhibited a robust, dose-dependent upregulation of both Nrf2 and HO-1. The data are presented as the mean ± SD (*n* = 3). Asterisks indicate statistically significant differences compared with the 0 μg/mL group (* *p* < 0.05, ** *p* < 0.01, *** *p* < 0.001).

To explore whether antioxidant-related transcriptional responses were associated with mud extract treatment, the mRNA expression levels of Nrf2 and its downstream effector HO-1 were analyzed using RT-qPCR. As shown in Figure 4A, mud extract treatment significantly induced Nrf2 mRNA expression in a dose-dependent manner. In RAW 264.7 macrophages, Nrf2 levels increased by 1.52-, 2.45-, and 4.12-fold at concentrations of 10, 100, and 1000 μg/mL, respectively, compared to the untreated control (0 μg/mL). In L929 fibroblasts, a significant increase in Nrf2 expression (1.32-fold) was observed only at the highest concentration (1000 μg/mL). The induction of HO-1 followed a similar but more robust pattern (Figure 4B). In RAW 264.7 cells, HO-1 mRNA expression was markedly upregulated, reaching a 6.45-fold increase at 1000 μg/mL (*p* < 0.001). L929 cells also exhibited a significant induction of HO-1 at 100 and 1000 μg/mL (1.28- and 1.48-fold, respectively). These findings support the association of mud extract treatment with antioxidant-related transcriptional responses, particularly in RAW 264.7 cells; however, they do not directly demonstrate protein-level activation or functional pathway completion.

## 4. Discussion

Oxidative stress, defined as an imbalance between ROS generation and antioxidant defense capacity, is a central process in cellular injury, inflammatory signaling, and tissue dysfunction [20,35]. In this context, natural mineral-rich materials such as therapeutic muds and peloids have long been used in balneotherapy and dermatologic applications, although the cellular mechanisms underlying their biological effects remain incompletely characterized [34,36,37,38]. The present study examined the effects of a mud extract in two murine cell lines with distinct biological roles, L929 fibroblasts and RAW 264.7 macrophages, by evaluating cell viability, intracellular ROS levels, antioxidant-related enzyme activities, and antioxidant-related gene expression. Overall, our findings indicate that the mud extract did not induce overt cytotoxicity under the tested conditions and was associated with antioxidant-related biochemical and transcriptional responses, particularly in RAW 264.7 cells. However, the present data should be interpreted as supportive of redox-associated modulation rather than as definitive proof of a fully established antioxidant mechanism. Importantly, this study was not designed to demonstrate direct clinical or industrial applicability, but rather to provide an initial in vitro assessment of redox-associated responses induced by a complex mud-derived material.

An important prerequisite for interpreting any antioxidant-related effect is confirmation that the tested material does not exert nonspecific cytotoxicity. In the present study, the mud extract did not cause substantial loss of viability in either L929 fibroblasts or RAW 264.7 macrophages up to 1000 μg/mL. This observation suggests that the tested concentration range remains within a cytocompatible window and supports the interpretation that subsequent redox-related findings are not simply secondary to overt structural or metabolic damage. This point is particularly relevant for mineral-rich materials, because apparent redox-related changes may otherwise reflect generalized cellular injury rather than biologically meaningful modulation [31,39,40,41]. At the same time, because these results were obtained under defined in vitro conditions using two murine cell lines, they should be interpreted as evidence of cytocompatibility within the present assay system rather than as a direct indicator of clinical safety or tolerability [42,43,44,45,46].

The ROS findings support a similarly cautious interpretation. Across the tested concentrations and time points, mud extract did not induce marked increases in intracellular ROS levels. In L929 fibroblasts, ROS levels remained close to untreated control throughout the observation period, whereas in RAW 264.7 macrophages, only modest reductions were observed at selected concentrations and time points. These results indicate that the extract did not trigger an overt oxidative burst under the present conditions. This is important because it argues against classifying the extract as a nonspecific oxidative stressor. At the same time, the current data do not support stronger interpretations such as hormesis, oxidative eustress, or a defined ROS-driven signaling response, because a clear transient ROS induction pattern was not observed. Moreover, DCFH-DA detects general intracellular oxidative activity rather than specific ROS species and is therefore best interpreted as a measure of relative oxidative status rather than pathway-specific redox signaling [10,47,48,49,50,51,52,53]. Accordingly, our ROS data are most appropriately interpreted as indicating the absence of overt oxidative disruption rather than direct evidence of a specific ROS-mediated regulatory mechanism.

The enzymatic assays provide additional support for antioxidant-related modulation, particularly in RAW 264.7 cells. In L929 fibroblasts, SOD-associated activity, CAT activity, and GPx activity remained relatively stable across concentrations and time points, suggesting that the mud extract did not induce a pronounced antioxidant response in this structural cell model. In contrast, RAW 264.7 macrophages exhibited a clearer temporal pattern. SOD-associated activity increased transiently at 1 h and then returned toward baseline by 6 h, whereas CAT and GPx activities increased at later time points, with the most prominent responses observed at 24 h. This pattern is compatible with a coordinated antioxidant-related response, in which early handling of superoxide is followed by increased downstream peroxide-detoxifying capacity [6,54,55,56,57]. Nevertheless, these findings should not be interpreted as definitive proof of direct enzymatic induction or a fully resolved antioxidant cascade. In particular, the SOD assay was based on WST chemistry, and although cell-free blank subtraction was used to reduce optical and matrix-related interference, residual assay dependency cannot be completely excluded. For this reason, the term “SOD-associated activity” remains more appropriate than a stronger mechanistic interpretation.

The transcriptional findings further support the biological relevance of the biochemical observations. Mud extract increased Nrf2 and HO-1 mRNA expression in a dose-dependent manner, particularly in RAW 264.7 macrophages, whereas L929 fibroblasts showed comparatively modest transcriptional changes. This pattern is consistent with activation of antioxidant-related transcriptional responses, because Nrf2 is a major regulator of antioxidant response element–driven genes and HO-1 is one of its representative downstream effectors [58,59,60,61]. In the present dataset, transcriptional induction at 12 h preceded the more prominent later increases in CAT and GPx activities, which is temporally compatible with the possibility that gene-level responses contribute to the subsequent biochemical profile [61,62]. However, these results should still be interpreted conservatively. The study did not assess Nrf2 nuclear translocation, HO-1 protein expression, or protein-level changes in downstream antioxidant enzymes. Therefore, the present qPCR findings provide supportive evidence for pathway involvement but do not establish direct pathway activation at the protein or functional level.

The stronger responses observed in RAW 264.7 cells than in L929 fibroblasts are also noteworthy. Macrophage-like cells are inherently more responsive to environmental stimuli and redox-sensitive perturbations than fibroblast-like structural cells because of their immunologic surveillance and inflammatory signaling functions [43,44,45,46,63,64,65,66]. The present findings are consistent with this biological distinction. RAW 264.7 macrophages exhibited clearer changes in antioxidant-related enzyme activities and more robust induction of Nrf2 and HO-1 transcripts, whereas L929 fibroblasts remained comparatively stable under the same treatment conditions. These observations suggest that the redox-associated effects of the mud extract may be cell type-dependent rather than universal across all cell populations. However, because the present study was not designed to define the mechanistic basis of these differences, this interpretation should remain descriptive. Additional studies comparing uptake kinetics, inflammatory signaling pathways, ionic responses, and stress-response thresholds across cell types would be needed to explain this divergence more directly [41,67,68]. In this context, the use of an aqueous mud extract represents an important interpretive context for this study. While extract-based approaches enable controlled in vitro evaluation of cellular responses, they do not fully capture the physicochemical complexity of the original material or the interactions among its components. Therefore, the observed effects should be interpreted as responses to a standardized extract fraction rather than direct representations of whole mud activity. Nevertheless, the use of dried-residue standardization and compositional profiling provides a reproducible baseline for future fractionation and mechanistic studies.

The elemental composition of the extract provides a biologically plausible context for the observed responses, but this aspect should also be interpreted with caution. The mud extract contained several inorganic elements, including Fe, K, Na, Mg, Ca, Mn, and Al. Some of these are broadly relevant to redox biology or antioxidant enzyme function. For example, Mn is associated with mitochondrial antioxidant systems, Fe is involved in heme-dependent and other redox-active cellular processes, and Mg and Ca contribute to intracellular signaling environments that may influence cellular stress adaptation [6,40,69,70,71,72,73,74,75,76,77]. These considerations make it plausible that the compositional profile of the extract contributed to the observed biochemical and transcriptional responses. However, the present study did not directly test the contribution of individual elements, determine their bioavailable intracellular fractions, or establish which components were causally responsible for the observed effects. Therefore, compositional data should be regarded as contextual support rather than direct mechanistic evidence.

Taken together, the present findings support the interpretation that mud extract may function as a non-cytotoxic redox-associated modulator in vitro rather than solely as a direct radical scavenger. The overall pattern observed here—absence of overt cytotoxicity, near-baseline ROS levels, temporally differentiated antioxidant-related enzyme responses in RAW 264.7 cells, and dose-dependent upregulation of Nrf2 and HO-1 transcripts—is consistent with coordinated antioxidant-related cellular adaptation under the tested conditions. However, the present data do not support stronger claims such as definitive antioxidant pathway activation, full restoration of redox homeostasis, or established therapeutic efficacy. The most defensible conclusion from the current evidence is that mud extract is associated with antioxidant-related biochemical and transcriptional responses under non-cytotoxic in vitro conditions.

Several limitations should be acknowledged. First, the study was performed exclusively in two murine cell lines and therefore cannot fully recapitulate the structural and immunological complexity of human tissues [43,44,45,46]. Second, some biochemical assays, particularly the WST-based SOD assay, may remain susceptible to residual matrix-related interference despite the use of cell-free blank subtraction. Third, intracellular ROS was assessed using a general fluorescent probe and therefore does not provide species-specific or pathway-specific information [10,47,48,49]. Fourth, the transcriptional analysis was limited to mRNA expression of Nrf2 and HO-1 and did not include protein-level validation or functional confirmation of pathway activation [58,59,60,61,62]. Finally, the present study did not identify which specific mineral components were responsible for the observed responses. Future studies should therefore incorporate orthogonal enzyme validation methods, protein-level analyses, primary human dermal or immune cells, and in vivo models to better define the mechanistic specificity, physiological relevance, and translational applicability of mud extract [34,36,37,38]. In addition, extract-based systems may be subject to batch-to-batch variability and compositional heterogeneity, which can limit reproducibility and generalizability. Future studies should address these aspects through fractionation approaches, component-specific analyses, and batch validation.

In conclusion, the present study shows that a standardized mud extract does not induce overt cytotoxicity and is associated with cell type-dependent redox-associated biochemical and transcriptional responses in vitro. These findings should be interpreted as exploratory evidence obtained under defined extract-based conditions rather than as proof of a definitive mechanism or immediate applicability. Further studies are required to clarify reproducibility, component-specific contributions, and translational relevance in more physiologically relevant systems.

## Figures and Tables

**Table 1:** Elemental composition of the mud extract determined by ICP-OES.

Element	Content (wt.%)
Al	7.59
Fe	2.87
K	2.52
Na	1.87
Mg	0.85
Ca	0.79
Mn	0.032

Contents are expressed as weight percentages (wt.%) relative to the dried-extract equivalents, which were obtained by lyophilization of the liquid extract.

## Data Availability

The data presented in this study are available from the corresponding author upon reasonable request. The raw experimental data supporting the conclusions of this article will be made available by the authors without undue reservation.
